# Search Engine Queries for Pediatric Fever During "Cold and Flu" Season

**DOI:** 10.7759/cureus.4464

**Published:** 2019-04-16

**Authors:** Joshua D Niforatos, Richard Pescatore

**Affiliations:** 1 Emergency Medicine, Cleveland Clinic Lerner College of Medicine, Case Western Reserve University, Cleveland, USA; 2 Emergency Medicine, Crozer Keystone Health System, Chester, USA

**Keywords:** pediatric fever, google trends, health literacy, search engine queries

## Abstract

Introduction

Pediatric fever is the most common chief complaint in patients under 15 years old. The objective of this paper is to characterize public search trends for pediatric fever in the United States using Google search engine queries.

Methods and materials

A cross-sectional survey of Google Trends searches for "toddler fever" was conducted from October 2018 to January 2019 during "cold and flu" season. Information collected included “Related Topics” and “Related Queries”, which includes additional searches by individuals who searched for “toddler fever”. Data are described in the results using Google’s relative popularity.

Results

For this study, 91 weeks of data were queried. The median relative popularity over this time period was 65 (interquartile range, 58 - 74.5) out of 100. Individuals searching for this term also searched thematically for characterizations and descriptors of fever, types of symptoms associated with fever, and various treatments for fever.

Conclusion

The results of this study revealed an increased frequency of search engine queries for descriptors and qualifiers of symptoms associated with pediatric illness during the "cold and flu" season. Frequently queried terms suggest a need for increased health literacy regarding pediatric fever in the United States and may represent a need for further national educational resources.

## Introduction

Pediatric fever is the most common chief complaint in patients under 15 years old presenting to the emergency department [1]. There is a dearth of literature on the public knowledge of pediatric fevers in the United States [2]. Past studies were primarily single-institution survey studies that identified knowledge related to the definition of fever and use of analgesics and antipyretics as insufficient [2]. Internet search queries, such as Google Trends [3] and Twitter [4] are frequently used as a form of public health ‘surveillance’ and 'infodemiology' [5-6]. For example, a recent study utilized geo-referenced social media near a pediatric hospital and found a positive correlation between tweets involving symptoms related to respiratory illness and emergency department volume related to influenza-like illness during the winter months [4]. More commonly in the literature, however, Google Trends (GT) is utilized for infodemiology [6].

GT is a publicly available tool from Google, Inc. that allows users to analyze trends of daily Google Search searches [3]. Previous studies utilizing GT have assessed internet searches for suicide [7], for sunburn as a surrogate marker for state sunburn prevalence [8], for vaccine-related information and concurrent searches by end-users [9], and more. Germane to pediatric fever, GT may provide more generalizable results compared to survey studies regarding questions asked by the public related to pediatric fever across the United States. Thus, in this study we sought to ascertain real-time trends in search engine queries related to pediatric fever during ‘cold and flu’ season in the United States.

## Materials and methods

We surveyed Google Search queries (https://trends.google.com) in the United States from October 13, 2018 to January 6, 2019. GT provides publicly available information of the relative popularity of queried terms by total searches in a geographic region over a specified period of time. GT provides this data as a frequency from 0 to 100 with 100 representing peak popularity of the term. We queried the term “toddler fever” using all search categories on January 13, 2019, during which Google’s search engine market share of all search engines was approximately 86.9% [10]. Information collected included “Related Topics” and “Related Queries”, which includes additional searches by individuals who searched for “toddler fever”. Queries represent matches for all related searches to “toddler fever”, while Topics represents a group of terms that share the same concept as “toddler fever”. Terms within Related Topics and Related Queries were categorized into (1) the most frequently searched concurrent terms (Top Terms) with "toddler fever" and (2) terms with the largest increase in concurrent search frequency (Rising Terms) over the given time period. Data are described in the results using Google’s relative popularity described above. This study of publicly available, de-identified data did not require Institutional Review Board approval.

## Results

We extracted 91 weeks of data related to the query “toddler fever”. The median relative popularity of this term over this time period was 65 (interquartile range, 58 - 74.5) out of 100, representing increased search activity for this term during the inclusion time period.

Table 1 characterizes Related Topics and Related Queries, which are classified as concurrent search terms made by end-users searching for "toddler fever".

**Table 1:** Google Searches Associated with “Toddler Fever” in the United States Abbreviations: RSV, Relative Search Volume RSV is reported as a frequency from 0 to 100 with 100 representing peak popularity of the term. "Increase" represents the largest increase in search frequency over the inclusion time period compared to all concurrent searches with "toddler fever". "Break out" represents an increase in search frequency by greater than 5,000% over the inclusion time period.

Related Topics	Related Queries

Top Searches	RSV	Rising Searches	Increase (%)	Top Searches	RSV	Rising Searches	Increase (%)
Toddler	100	Tremor	Breakout	Fever in toddler	100	100.6 fever in toddler	Breakout
Fever	99	Contagious disease	Breakout	Toddler with fever	64	Fever temperature chart	300%
Cough	13	Apple cider vinegar	Breakout	High fever toddler	29	Toddler vomiting no fever	150%
Vomiting	11	Eye pain	Breakout	Fever rash toddler	20	Toddler puking no fever	90%
Symptom	10	Suppository	Breakout	High fever in toddler	15	Fever and cough in toddler	60%
Influenza	7	Bronchitis	Breakout	Toddler fever temperature	13		
Common cold	7	Influenza	400%	Toddler fever 102	12		
Skin rash	6	Human orthopneumovirus	350%	Low grade fever	10		
Breathing	5	Celsius	250%	Fever in toddlers	9		
Diarrhea	5	Sore throat	200%	Toddler throwing up no fever	9		
Infection	4	Tachypnea	160%	Toddler vomiting no fever	9		
Temperature	4	Cough	150%	Toddler fever no other symptoms	8		
Rhinorrhea	3	Nasal congestion	130%	What is a fever for a toddler	7		
Sleep	3	Common cold	90%	High fever for toddler	7		
Nasal congestion	2	Gastroenteritis	80%	Fever and cough in toddler	7		
Night	2	Sleep	70%	104 fever toddler	4		
Ibuprofen	2	Diarrhea	70%	Toddler fever chart	3		
Celsius	2	Rhinorrhea	70%	Low grade fever in toddler	3		
Tachypnea	2	Fahrenheit	60%	Rash and fever in toddler	2		
Abdomen	2	Vomiting	50%	Toddler fever when to worry	2		
Streptococcal pharyngitis	2	Breathing	50%	Roseola	2		
Hand	2			When to take toddler to doctor for fever	2		
Month	2			Fever temperature chart	1		
Fahrenheit	2			Toddler throwing up with no fever	1		
Otitis	2			Toddler puking no fever	1		

Individuals searching for this term searched thematically (Topics) for fever, symptoms (cough, vomiting, tremor, eye pain, sore throat, tachypnea, nasal congestion), and treatments (apple cider vinegar, suppository, Ibuprofen) (Table 1). Additionally, searches (Queries) included variations of “toddler fever” (fever in toddler, toddler with fever), as well as descriptors of fevers (high fever, fever 102, 100.6 fever, low grade fever, 104 fever, what is a fever for a toddler) and additional symptoms (rash, no other symptoms, cough). Queries also included emesis in the absence of fever.

The "Increase" column in Table 1 for both "Rising Topics" and "Queries" represents the largest increase in search frequency over the inclusion time period compared to all concurrent searches with "toddler fever". End-users searching for "toddler fever" were increasingly searching for symptoms, such as tremor, eye pain, sore throat, and cough, as well as if "toddler fever" is "contagious". 

## Discussion

The results of this study highlight an increased search engine queries related to pediatric (toddler) fever during "cold and flu" season, often coupled with descriptors or qualifiers (particularly, height of fever), and concomitant symptoms. These findings corroborate the recent extensive systematic review and meta-analysis (SRMA) by Bertille et al. [2] that investigated parents' knowledge and behaviors of pediatric fever internationally from 1980 to 2016. More specifically, this SRMA revealed that parents' knowledge and behaviors of definitions of pediatric fever (≥ 38°C) had changed over time, though the findings were non-significant and showed poor concordance with national recommendations [2]. Bertille et al. [2] suggest the reasons for such misunderstanding of pediatric fever may be secondary to "the low readability of patient educational tools, poor dissemination of guidelines [11] and incorrect or suboptimal counseling and examples provided by healthcare providers" [12]. Our study adds to this literature by providing real-time data during "cold and flu" season that suggests the public continues to search for basic definitions of fever. 

The results of our study, however, should be interpreted with caution and have several limitations. The use of GT in this study did not allow for causal assessment regarding whether search engine queries are secondary to increased or lack of health literacy; however, it would be unusual for those with increased health literacy to search for basic definitions of fever. Additionally, while this study is limited to people who use Google for their web-based browsing (and who represent almost 90% of all end-users of the Internet during this study [10]), as well as reliance on relative popularity instead of volume of searches, the search activities observed in this study represent a need for further national educational resources on this topic [13]. Previous studies have demonstrated that, e.g., parental lack of knowledge of pediatric health is significantly associated with increased emergency department utilization [4]. Efforts designed to increase health literacy may benefit from observation of search trends to design electronic resources and curricula-on the Internet, mobile apps for smartphones, etc.‚-that address the most commonly searched themes related to febrile illness in children. 

## Conclusions

The results of this study revealed increased frequency of search engine queries for descriptors and qualifiers of symptoms associated with pediatric illness during "cold and flu" season. Frequently queried terms, such as "100.6 fever in toddler" and "fever in toddler", suggest a need for increased health education regarding febrile illness in children in the United States. Given that pediatric fever is the most common chief complaint in patients under 15 years old presenting to the emergency department, continued development for further national educational resources on basic definitions of pediatric fever, as well as symptoms associated with fever that warrant medical attention, are required. 
